# The role of defects in organic image sensors for green photodiode

**DOI:** 10.1038/s41598-018-36105-9

**Published:** 2019-02-11

**Authors:** Seong Heon Kim, Jooho lee, Eunae Cho, Junho Lee, Dong-Jin Yun, Dongwook Lee, Yongsung Kim, Takkyun Ro, Chul-Joon Heo, Gae Hwang Lee, Yong Wan Jin, Sunghan Kim, Kyung-Bae Park, Sung Heo

**Affiliations:** 1 Platform Technology Lab, Samsung Advanced Institute of Technology, 130, Samsung-ro, Yeongtong-gu, Suwon-si, Gyeonggi-do, 443-803 Korea; 2 Organic Materials Laboratory, Samsung Advanced Institute of Technology, 130, Samsung-ro, Yeongtong-gu, Suwon-si, Gyeonggi-do, 443-803 Korea

## Abstract

Controlling defect states in a buffer layer for organic photo devices is one of the vital factors which have great influence on the device performance. Defect states in silicon oxynitride (SiO_x_N_y_) buffer layer for organic photo devices can be controlled by introducing appropriate dopant materials. We performed *ab initio* simulations to identify the effect on doping SiO_x_N_y_ with carbon (C), boron (B), and phosphorous (P) atoms. The results unveil that hole defects in the SiO_x_N_y_ layer diminish with the phosphorous doping. Based on the simulation results, we fabricate the small molecule organic photodetector (OPD) including the phosphorous-doped SiO_x_N_y_ buffer layer and the active film of blended naphthalene-based donor and C60 acceptor molecules, which shows excellent enhancement in the external quantum efficiency (EQE). The results of our charge-based deep level transient spectroscopy (Q-DLTS) measurements confirmed that the EQE enhancement originates from the decrease of the hole traps induced by the reduced hole defects. The method of controlling the defect states in SiO_x_N_y_ buffer layers by the doping can be used to improve the performance in various organic photo devices.

## Introduction

Growing need for high resolution sensors in imaging devices has strongly demanded new device schematic concepts to improve the resolution of conventional complementary metal–oxide–semiconductor (CMOS) color image sensors (CISs). One solution is to stack the structure 3-dimensionally, increasing the effective light detection area per each pixel. Color filters are indispensable in the conventional Si-based CISs for the selective detection of red (R), green (G), and blue (B) colors because the broad absorption band of Si exists in the wide wavelength range of 400~700 nm. Typically, they include two-dimensional color filter arrays of R, G, and B pixels, i.e., the Bayer filter pattern^1^. Therefore, it is difficult to apply the 3D stacking structure concept in conventional Si-based CISs. As an alternative, organic materials can be a great solution to overcome the limit of Si-based CISs because they have intrinsic narrow absorption bands, indicating that color filters are not necessary for the selective R, G, and B color detection by using organic materials. In addition, a great number of organic molecules for imaging electronics have been already developed and new organic molecules can be engineered in accordance with the purpose. Thus, it is possible to choose organic materials selectively for R, G, and B colors in organic-based CISs. In this paper, we used the green image sensor which has been applied in the organic-on-Si hybrid CIS developed in Samsung advanced institute of technology (SAIT)^2^. In the organic-on-Si CIS, the effective light detection area per pixel is doubled by stacking a G-light selective organic photodetector (OPD) on a Si-based CMOS circuit containing B and R color filters^2,3^.

There has been a plenty of effort to improve the device performances of OPDs including external quantum efficiency (EQE), dark current (DC), and thermal stability, which are the key factors to evaluate the performance of OPDs^4–13^. EQE is coupled to the efficiency and signal-to-noise ratio (SNR) of the devices. Low DC stabilizes the signal of the devices, leading to high SNR. Because organic materials in OPDs are exposed to high temperature process such as post-annealing, passivation step, top layer planarization, and microlens forming process, they should be thermally stable without performance degradation. Appropriate buffer layers such as MoO_x_, WO_x_, VO_x_, and triphenylamine derivative (TPD) has been introduced in the OPD fabrication for decreasing DC and enhancing thermal stability^14–18^. However, it is very difficult to change the band structure of these materials (MoOx, WOx, VOx, and TPD), i.e. their highest occupied molecular orbital (HOMO) and lowest unoccupied molecular orbital (LUMO) levels do not change depending on the subsequent active materials. Therefore it is necessary to develop new buffer layers whose band structure can be controlled with ease. In our previous paper, the silicon oxynitride (SiO_x_N_y_, SiON) layer has been introduced as a good buffer layer for OPDs because its band structure can be freely tuned by adjusting the atomic concentration of oxygen (x) and nitrogen (y)^19^. By controlling the x and y, the band structure of SiO_x_N_y_ buffer layer can be aligned well to HOMO and LUMO levels of bulk heterojuction (BHJ) films. In the OPD devices using the SiO_x_N_y_ buffer layer, the detectivity and the thermal stability were improved as well as the DC decreased^19^. Here, we propose a novel approach to improve the OPD performance by controlling the defect states within the energy band gap. Our *ab initio* simulations demonstrate that the defect states of SiO_x_N_y_ buffer layer are controlled by doping with various atoms such as C, B, and P. In addition, we could achieve excellent enhancement in EQE of our OPD device including the SiO_x_N_y_ buffer layer which had reduced defect states by doping with P atoms.

**Figure 1:**
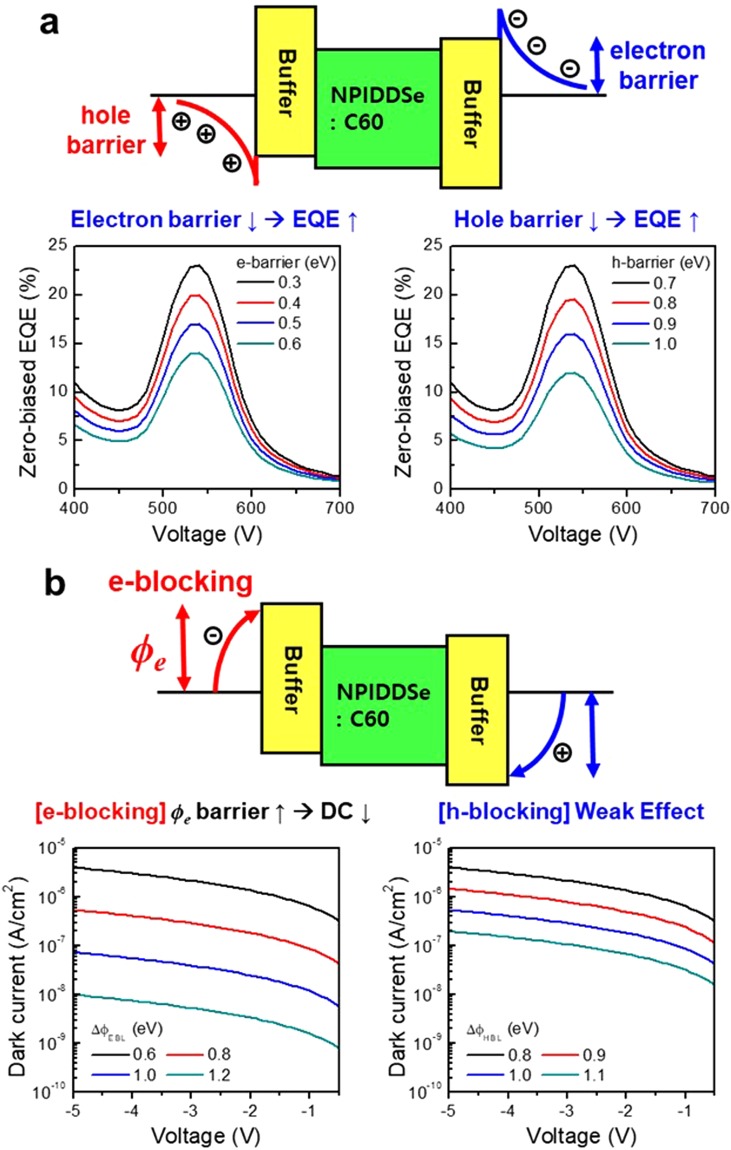
(**a**) EQE and (**b**) DC performance depending on the characteristics of the buffer layers in the device structure of ITO/EBL/OPD/HBL/ITO, calculated using the SETFOS software tools.

The energy band gap of the buffer layer is a crucial factor which has great influence on the device performance, especially, EQE and DC. As shown in the simulation results (Fig. 1) using the SETFOS software tools, we calculated the device performance (EQE, DC) depending on the characteristics of buffer layers, electron blocking layer (EBL) and hole blocking layer (HBL), in the device structure of ITO/EBL/OPD/HBL/ITO. As shown in Fig. 1a, EQE increases as the hole barrier in EBL or the electron barrier in HBL gets lower, i.e. HOMO of EBL or LUMO of HBL decreases. In addition, as shown in Fig. 1b, DC decreases as the electron blocking of EBL increases, i.e. LUMO of EBL decreases, while it is not significantly influenced on the hole blocking of HBL, i.e. HOMO of HBL.

The other key factor for the OPD device performance is to adjust the density of the defect states within the band gap of the buffer layer^10^. In most cases, defect states can act as a trap for electrons or holes, which interrupts their transport and decreases EQE. Therefore, one of the crucial issues for developing high performance OPDs is to control the defect states within the bandgap of the buffer layer. To verify the change of defect states within the bandgap of SiO_x_N_y_ by the doping, we carried out *ab initio* calculations using the Vienna *ab initio* Simulation Package (VASP) and molecular dynamics simulations for amorphous phase modeling^20^. The atomic and the electronic structure of amorphous phase were obtained by calculating the structural properties such as radial distribution function and angle distribution function by the melt-quench method of molecular dynamics simulations and the projector-augmented-wave (PAW)-generalized gradient approximation (GGA) potential^21,22^ The electrical properties including inverse participation ratio, density of states, wave function and so on were also calculated. The energy cutoff was chosen to be 400 eV and 2 × 2 × 1 Monkhorst-Pack k-point meshes were used.

The possible defect states in amorphous SiN were also calculated. The results indicate that k-defects (Si-dangling bond), Si-Si homopolar bonds, under-coordinated N atoms, and distorted bonds are the main defects in SiN. Particularly, dangling bonds of Si or N, and unstable Si-Si bonds induce localized defect states near the valence band. The distorted bonds create the defect states near the conduction band, having an influence on the leakage current.

In addition, over-coordinate Si atoms (5-fold Si, 6-fold Si) make defect levels near the valance band and over-coordinated N atoms (4 fold N) make localized defect levels near the conduction band (Supplementary Fig. S1).

**Figure 2:**
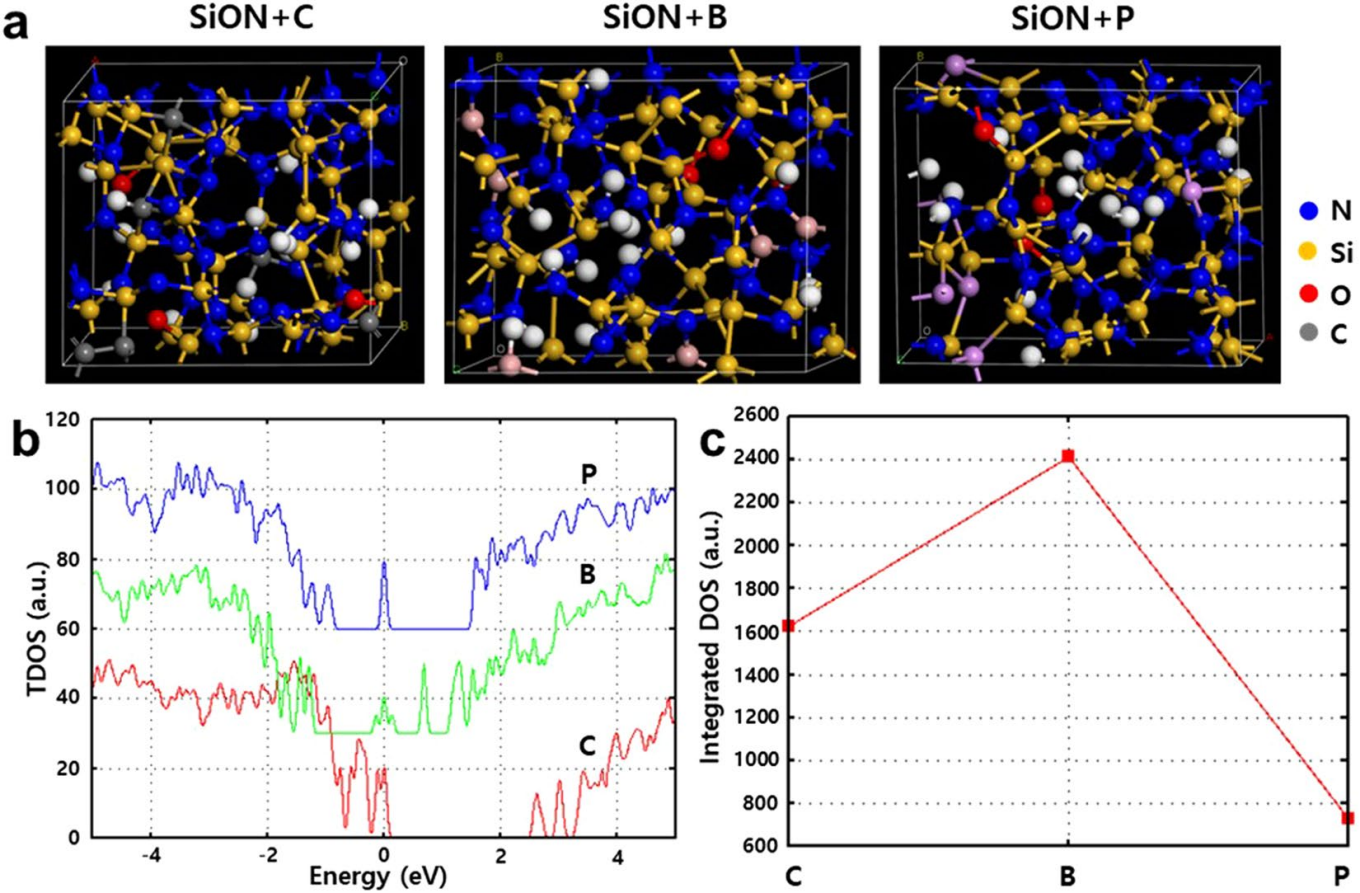
Results of *ab initio* calculations. (**a**) Structural change of SiON by C-, B-, P-doping. (**b**) Calculated electronic structure of SiON and (**c**) plot of integrated electron density of states for C-, B-, P-doping.

As shown in Fig. 2, to verify the doping effects on SiON, we analyzed the geometric and the electronic structure of SiON with changing the dopant materials (C, B, P). First, in the case of changing the C content from 2 to 6 at.%, C atoms reduced the Si-Si bonds by forming the bond with Si and reduced also the distorted bonds by inducing tetrahedral bonds. As a result, the addition of C atoms of 2~6 at.% induced the reduction of defects. However, the addition of C atoms more than 6% increased defects by forming C-C clusters which induced the defect states in the band gap. In addition, we performed the calculations for adding B and P atoms of 6 at.%. In the case of B atoms, they formed the bond with N and induced the reduced C-Si or N-Si bonds, which resulted in the increased Si-Si homopolar bonds. This increased the band gap and defect states. In the case of P atoms, they mainly formed the bonds with Si atoms and contributed to the reduction of Si-dangling bonds in the system, which induced the reduction of defects. The results of these calculations for the three kinds of atoms (C, B, and P) confirmed that P-doped SiON has the lowest defect density, while B doping increases the defect density at maximum level (Fig. 2, Table 1).

**Table 1:** Atomic structure (bond number): SiNOH + X (X = B, P, C).

	Partial bond number for H tom		
	C	B	P
Si	2.83	0.51	1.46
N	0.21	2.67	0.33
O	0	0	0.17
H	0.66	0.16	0.12
Total	3.71	3.37	2.43
(Si-Si)/(Si-X)	3.7	10	2.8
Si dangling bond	2	10	2
N dangling bond	1	2	0

Based on the simulation results, doping of the SiON films with C and P atoms were carried out and their electrical properties were characterized. (Supplementary Fig. S2).

As shown in Fig. S2, the leakage current increases in the C-doped sample. However, the P-doped sample shows the lower breakdown voltage by comparing with the reference un-doped SiON sample. The results indicate the reduction of defects by the P-doping.

## Results and Discussion

**Figure 3:**
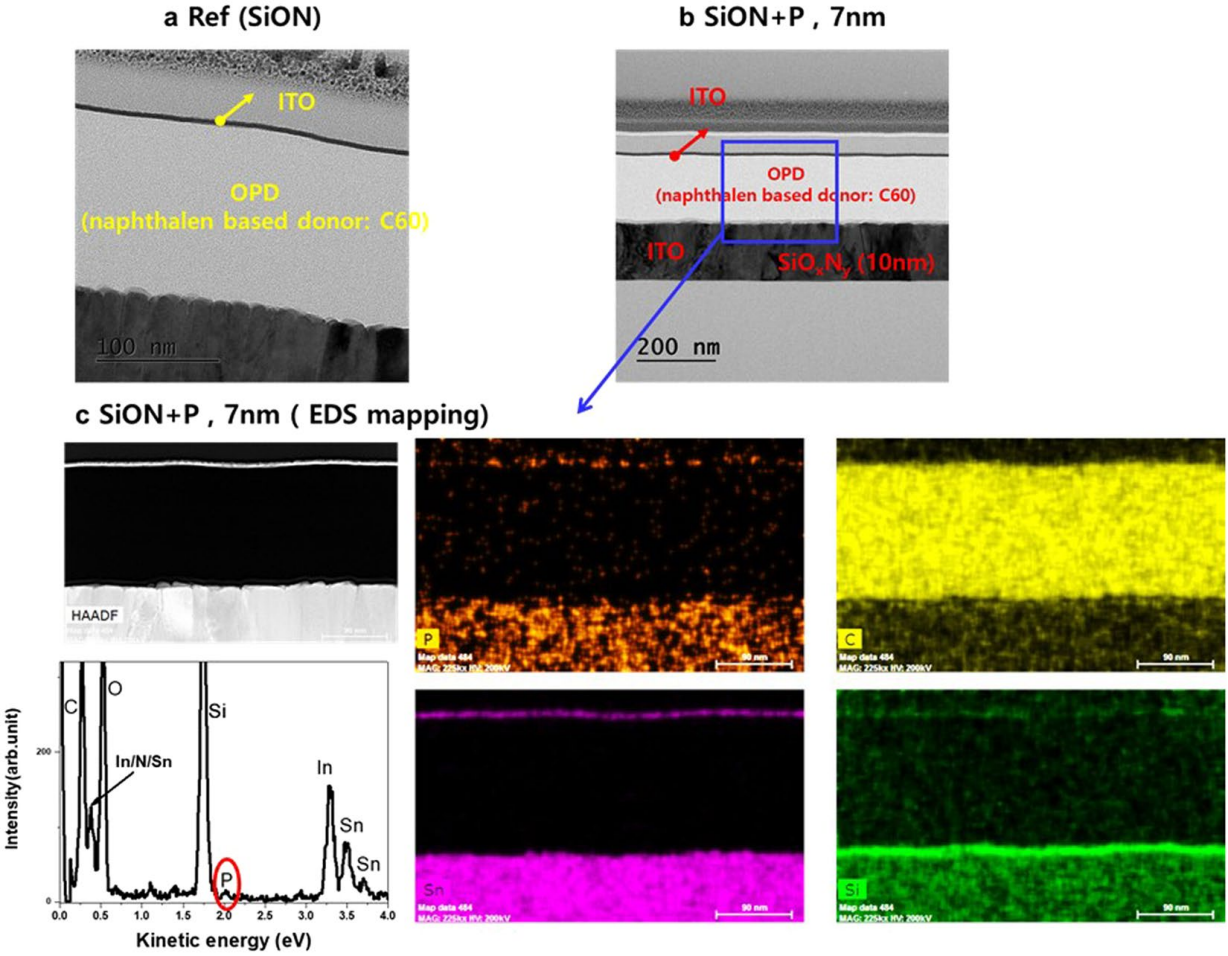
(**a**,**b**) Cross sectional TEM image of (**a**) reference (SiON film) and (**b**) SiON + P (7 nm). (**c**) EDS elemental mapping of SiON + P (7 nm) sample in (**b**).

**Figure 4:**
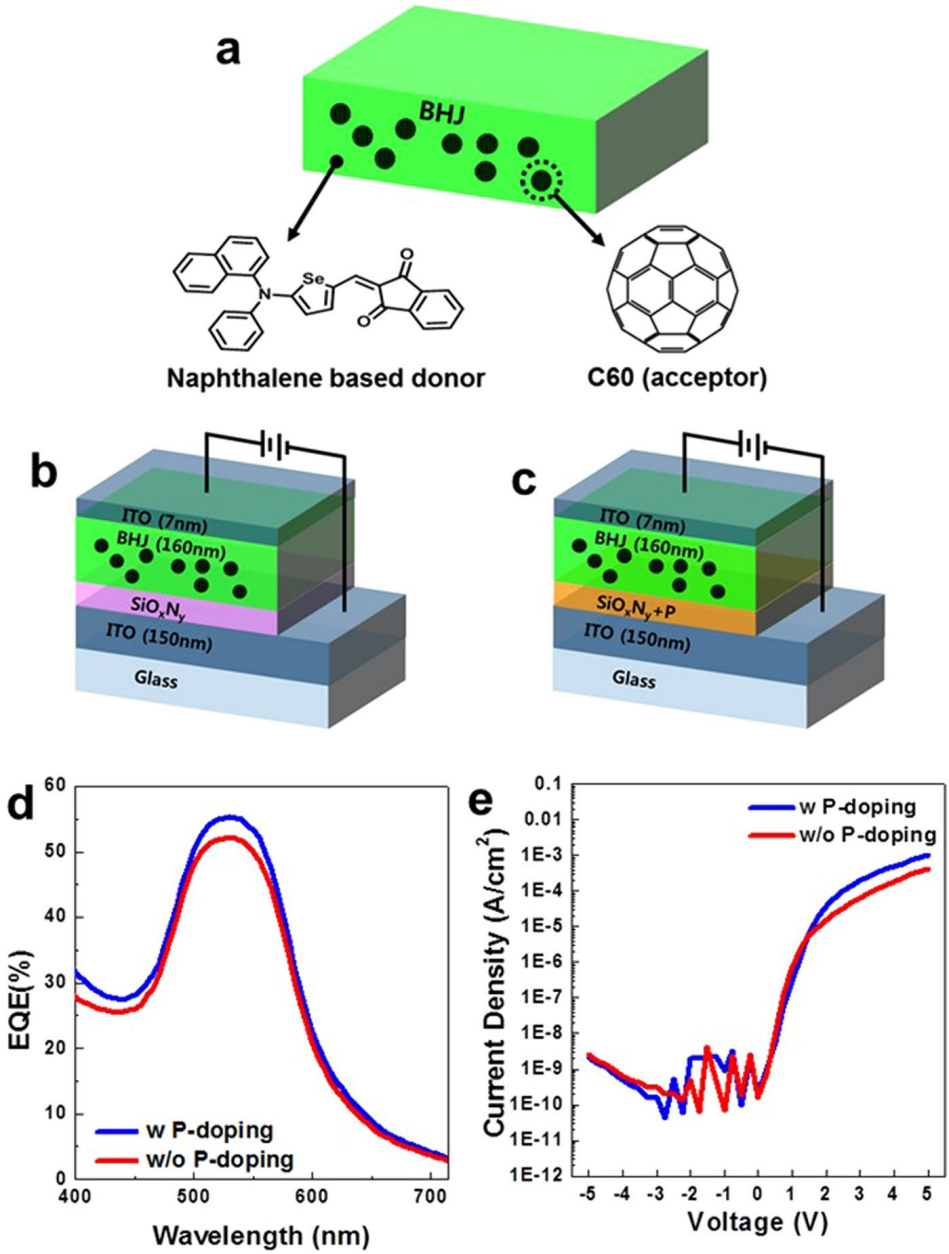
(**a**) Structure of BHJ active layer consisting of 1:1 blended naphthalene- based donor and C60 acceptor molecules. (**b**), (**c**) Structure of OPD devices including (**b**) pristine and (**c**) P-doped SiO_x_N_y_ buffer layers. (**d**) EQE and (**e**) DC characteristics for OPDs including the pristine and the P-doped SiOxNy buffer layers.

To verify the effect on the OPD device performance by P-doping in the SiO_x_N_y_ buffer layer, we fabricated the OPD devices with the P-doped SiO_x_Ny buffer layers. The cross-sectional transmission electron microscopy (TEM) images for our OPD devices and their elemental maps by energy-dispersive X-ray spectroscopy (EDS) are shown in Fig. 3. Each OPD device consist of a buffer layer of SiO_x_N_y_ (7 nm) and an active layer (160 nm) of 1:1 blended naphthalene-based donor and C60 acceptor molecules, as depicted in Fig. 4a–c. Two kinds of buffer layers, the reference and the P-doped SiO_x_N_y_, were prepared. The Si-rich SiO_x_N_y_ films were sequentially deposited on indium tin oxide (ITO)-coated glasses by plasma-enhanced chemical vapor deposition (PECVD) using various SiH4:NH3:N:PH3 gas mixtures with carrier N2 gas; 670 W of RF power was applied and the deposition temperature was 180 °C. The doping level of phosphorous was performed by controlling the PH3 gas flow from 0 to 180 sccm. The ratios of x (O/Si) and y (N/Si) are 0.10 and 0.88, respectively.

The ratios of x (O/Si) and y (N/Si) are 0.10 and 0.88, respectively. The reference sample (Fig. 4b) has a pure SiO_x_N_y_ buffer layer without doping, while the P-doping sample (Fig. 4c) was applied for the SiO_x_N_y_ buffer layer of the doped sample.

After the preparation of pure and P-doped SiO_x_N_y_ buffer layers, respectively, a 160-nm-thick organic bulk hetero junction (BHJ) layer, which is the 1:1 blend film of ‘naphthalene based donor’ and C60, was formed on the SiO_x_N_y_ layers, followed by the deposition of a 7-nm-thick ITO capping layer. The ‘naphthalene based donor’, shown in Fig. 4a, is a novel push-pull-structured organic semiconducting material, which is 5- (naphthalen-1-yl (phenyl) amino) selenophen-2-yl) methylene) -1H-indene-1,3 (2 H) -dione, with the absorption properties selective to green-light and it has a LUMO and HOMO values as a 3.6 eV and 5.5 eV as respectively.

The device performances of the OPD samples such as EQE and DC were characterized and were shown in Fig. 4d,e. The EQE of the sample with a P-doped SiO_x_N_y_ buffer layer increased by ~3.4%, while the DC characteristic of the P-doped sample is similar to that of the reference one. In Fig. 4e, it is notable that both samples with SiO_x_N_y_ layers show the improved DC performance regardless of P-doping, in comparison with the OPD sample without a SiO_x_N_y_ buffer layer.

To elucidate the mechanism of enhanced EQE characteristic by the P-doping of SiO_x_N_y_ buffer layer, we performed the charge based deep level transient spectroscopy (Q-DLTS) measurements for the two OPD samples, in which the temperature is kept constant while the frequency is scanned. This method has been particularly successful to measure the current transient for organic materials^23–25^.

**Figure 5:**
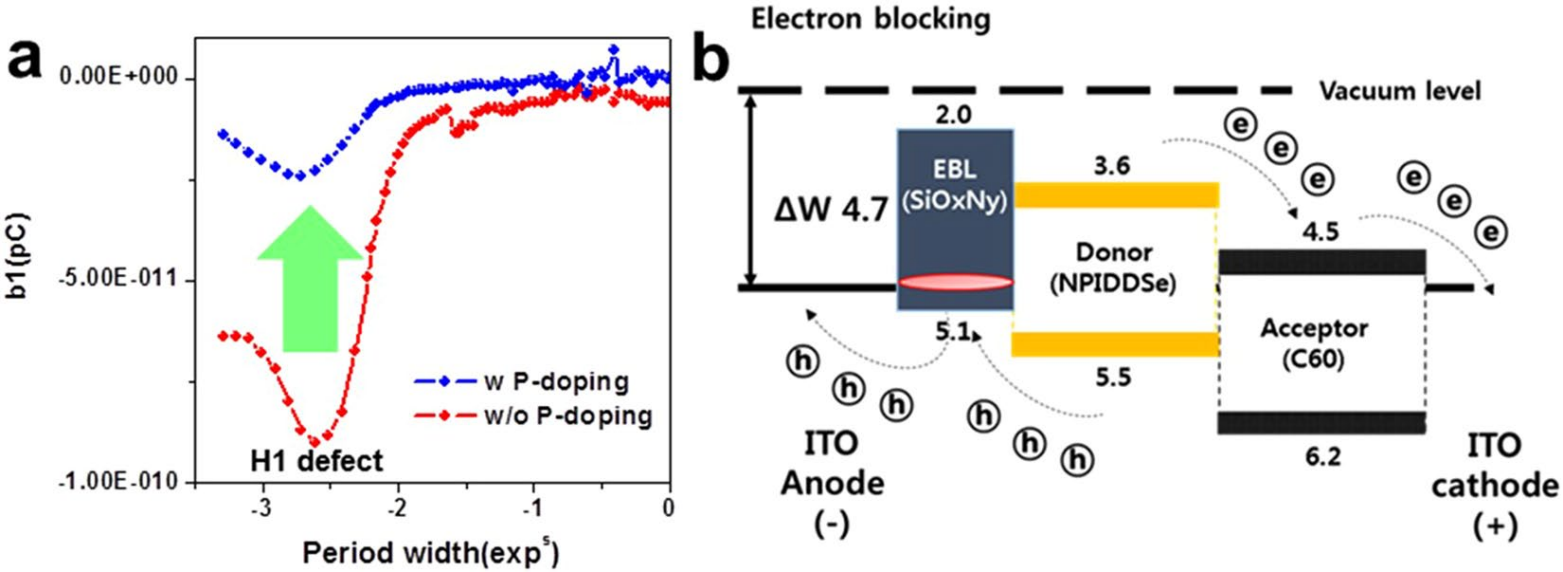
(**a**) Q-DLTS results for OPDs including the pristine and the P-doped SiO_x_N_y_ buffer layers. (**b**) Mechanism of improved EQE performance by P-doping.

In good agreement with our simulation results, the hole defect states near the energy level, which is 0.3 eV higher than the valence band energy level, remarkably decreases by the P-doping of SiO_x_N_y_ buffer layer, as shown in Fig. 5a. Figure 5b depicts the mechanism for the enhanced EQE by the reduction of hole defect states. The holes and electrons separated from excitons should be transported to the ITO electrodes without being disturbed to obtain the high performance in EQE. However, the hole defect states in the SiO_x_N_y_ buffer layer can act as the hole traps which interrupt the transport of holes, as shown in Fig. 5b.

## Conclusions

In this study, for the purpose of controlling the defect states of SiO_x_N_y_ buffer layer for OPDs, we performed the *ab initio* simulations for the doping of SiO_x_N_y_ with C, B, and P. It was revealed that the hole defects in SiO_x_N_y_ could be reduced by P doping. In the device performance measurements, the OPD with a P-doped SiO_x_N_y_ buffer layer shows the improved EQE. Based on our QDLTS measurements, it was experimentally confirmed that the EQE enhancement originates from the reduced hole traps induced by the reduced hole defects. The method to control the defect states in SiO_x_N_y_ buffer layers by the doping can be used to improve the performance in various organic photo devices.

## Methods

### Characterizations

Transmission electron microscopy (TEM) imaging and energy-dispersive X-ray (EDX) analysis were performed on a FEI Tecnai Osiris at 200 kV. This microscope was equipped with a high brightness X-FEG gun and silicon drift Super-X EDX detectors and Bruker Esprit acquisition software.

The composition analysis and valance band offset measurements was carried out by X-ray photoelectron spectroscopy (XPS, PHI Quantera II Scanning XPS Microprobe).

The HR-REELS investigation was conducted by Auger electron spectroscopy (AES; PHI 4700 system) with attached monochromatic electron gun (LK 1000). The primary energy of monochromatic electrons for the HR-REELS measurement was 0.3 keV and the full width at half maximum (FWHM) of the elastic peaks of 0.3 keV was 50 meV.

Q-DLTS measurements were performed with a PhysTech FT 1030 DLTS system. Temperature scans were made between 300 and 350 K, at a heating rate of 5 K/min. Samples were placed in the liquid helium cryostat. The pulse height, filling pulse width, and pulse period width were 0.4 V, 10 ms, and 10 ms, respectively. Here, reverse bias is −0.8 V to −0.4 V, and forward bias is 0.4 V to 0.8 V, respectively. The activation energy, capture cross-section, and concentration of traps were calculated using an Arrhenius plot.

## Electronic supplementary material

SUPPLEMENTARY INFO
