# Supplementary material for: The role of defects in organic image sensors for green photodiode
Source: Sci Rep. 2019 Feb 11;9:1745. doi: 10.1038/s41598-018-36105-9 (PMC6370835; doi:10.1038/s41598-018-36105-9)
Supplement: Supplementary file 1 — SUPPLEMENTARY INFO [file 41598_2018_36105_MOESM1_ESM.docx]

**The role of defects in organic image sensors for green photodiode**

Seong Heon Kim^1†^, Jooho lee^1†^, Eunae Cho^1^, Junho Lee^1^, Dong-Jin Yun^1^, Dongwook

Lee^1^, Yongsung Kim^1^, Takkyun Ro^2^, Chul-Joon Heo^2^, Gae Hwang Lee^2^, Yong Wan Jin^2^, Sunghan Kim^2^, Kyung-Bae Park ^2*^, Sung Heo^1**^

*^1^ Platform Technology Lab, Samsung Advanced Institute of Technology, 130,*

*Samsung-ro, Yeongtong-gu, Suwon-si, Gyeonggi-do, Korea, 443-803*

*^2^ Organic Materials Laboratory, Samsung Advanced Institute of Technology, 130,*

*Samsung-ro, Yeongtong-gu, Suwon-si, Gyeonggi-do, Korea, 443-803*

^†^ Seong Heon Kim and Jooho lee contributed equally to this study

*E-mail: [myshkin.park@samsung.com](mailto:myshkin.park@samsung.com)

**E-mail : [prestine@hanmail.net](mailto:prestine@hanmail.net)


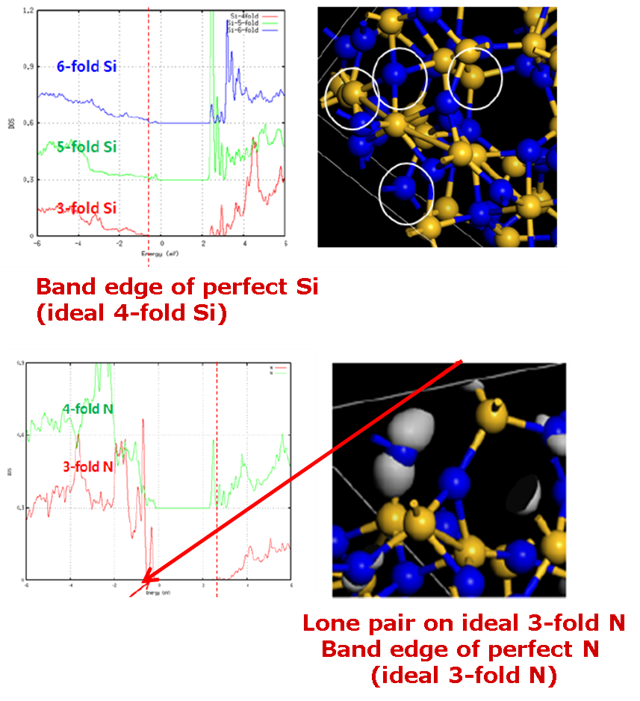


Figure S1. Density of state using the first principle calculation of (a) under/over coordinate number of Si (3 fold Si, 5 fold Si and 6 fold Si) (b) over coordinate number of N ( 3 fold N , 4 fold N)


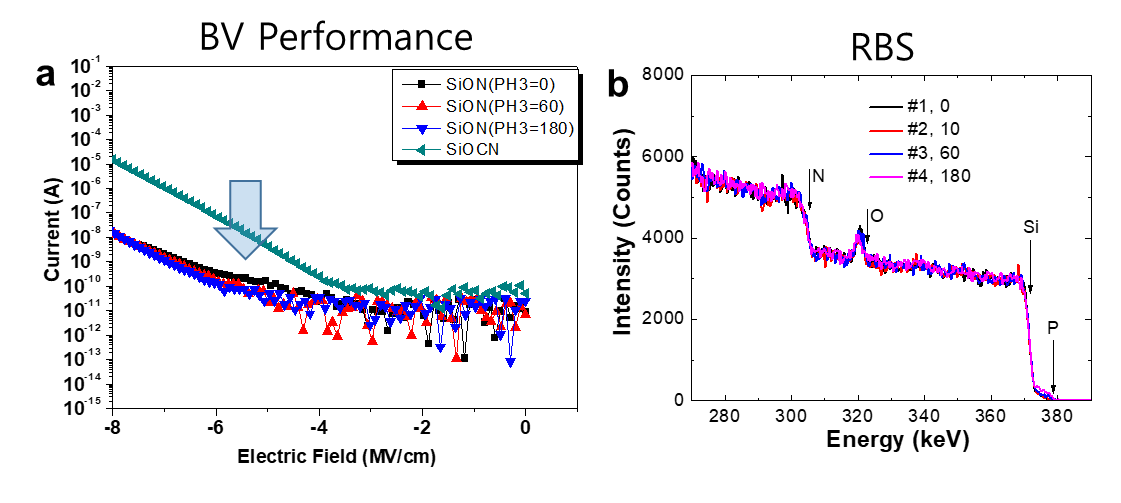


Figure S2. (a) Measurement of breakdown voltage in doping the SiON films with C and P atom. (b) RBS analysis of SiON film with various compositions of phosphorous atoms (ref, 60sccm, and 180sccm).
